# Supplementary material for: Intestinal stroma guides monocyte differentiation to macrophages through GM-CSF
Source: Nat Commun. 2024 Feb 26;15:1752. doi: 10.1038/s41467-024-46076-3 (PMC10897309; doi:10.1038/s41467-024-46076-3)
Supplement: Supplementary file 1 — Supplementary Information [file 41467_2024_46076_MOESM1_ESM.pdf]

Supplementary Information

Intestinal stroma guides monocyte differentiation to macrophages through GM-CSF

Egle Kvedaraite, Magda Lourda, Natalia Mouratidou, Tim Düking, Avinash Padhi, Kirsten Moll, Paulo Czarnewski, Indranil Sinha, Ioanna Xagoraris, Efthymia Kokkinou, Anastasios Damdimopoulos, Whitney Weigel, Olga Hartwig, Telma E Santos, Tea Soini, Aline Van Acker, Nelly Rahkonen, Malin Flodström Tullberg, Emma Ringqvist, Marcus Buggert, Carl Jorns, Ulrik Lindforss, Caroline Nordenvall, Christopher T Stamper, David Unnersjö-Jess, Mira Akber, Ruta Nadisauskaite, Jessica Jansson, Niels Vandamme, Chiara Sorini, Marijke Elise Grundeken, Helena Rolandsdotter, George Rassidakis, Eduardo J Villablanca, Maja Ideström, Stefan Eulitz, Henrik Arnell, Jenny Mjösberg, Jan-Inge Henter, Mattias Svensson

Correspondence: egle.kvedaraite@ki.se, mattias.svensson@ki.se

List of supplementary information

1. Supplementary Fig. 1: Evaluation of the level of inflammation in matched pediatric intestinal biopsies (related to Fig. 1).
2. Supplementary Fig. 2: Inflammation related changes in intestinal pediatric IBD stroma revealed by scRNA-seq (related to Fig. 2).
3. Supplementary Fig. 3: Composition of intestinal stroma and immune cells validated across colon segments and intestinal layers using 25-color flow cytometry (related to Fig. 2).
4. Supplementary Fig. 4: Ultra high content imaging MACSima (related to Fig. 3).
5. Supplementary Fig. 5: Ultra high content imaging reveals spatial composition of intestinal stroma and immune cells (related to Fig. 3).
6. Supplementary Fig. 6: Individual gene expression from monocyte/macrophage differentiation/recruitment score (related to Fig. 4g, h).
7. Supplementary Fig. 7: PDGFRA^{+}CD142$^{low/-}$ fibroblasts in vitro (related to Fig. 5).
8. Supplementary Fig. 8: PDGFRA^{+} fibroblasts guide monocyte to macrophage transition through GM-CSF (related to Fig. 5).
9. Supplementary Fig. 9: Double staining of intestinal PDGFRAB+ fibroblasts and GM-CSF (related to Fig. 5).
10. Supplementary Fig. 10: CSF-2 (GM-CSF) transcriptional levels (related to Fig. 5).
11. Supplementary Table 1: Clinical characteristics of all recruited patients.
12. Supplementary Table 2: Individual patient characteristics and laboratory data of a selected newly diagnosed patient cohort.
13. Supplementary Table 3: Antibodies.
14. Supplementary Table 4: Antibodies for ultra-high content imaging.

Supplementary Fig. 1

a Identification of intestinal neutrophils

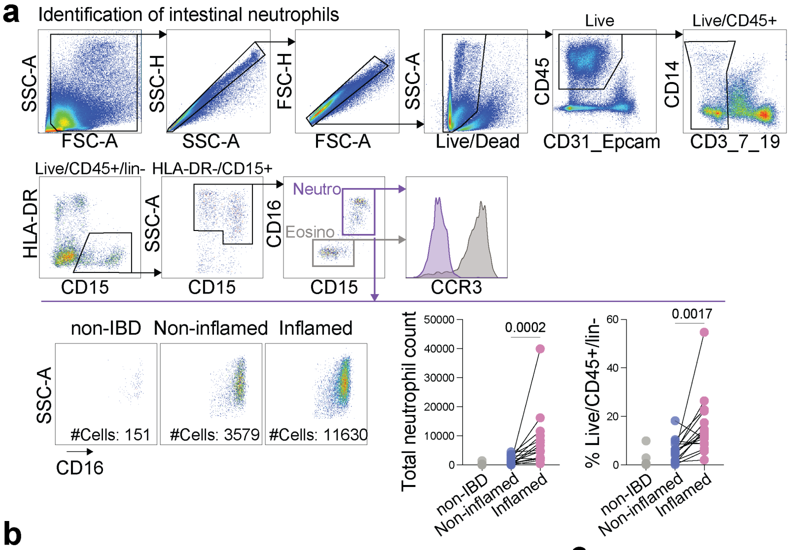

b

PATIENT	1. Macroscopic definition		2. Microscopic definition	3. Neutrophil counts
	Where the non-inflamed and inflamed biopsies were collected?		Were the non-inflamed area confirmed as strictly non-inflamed after the histopathology review?	Higher neutrophil counts confirmed in the inflamed compared to the non-inflamed area, defined by flow cytometry?
	Non-inflamed	Inflamed		
P01	ascendens	sigmoideum	YES	YES (inf = 2120, non-inf = 673)
P02	ascendens	descendens	YES	YES (inf = 7771, non-inf = 188)
P03	caecum	rectum	YES	YES (inf = 39948, non-inf = 872)
P04	flexura sin	sigmoideum	YES	YES (inf = 6705, non-inf = 47)
P05	caecum	sigmoideum	YES	YES (inf = 2325, non-inf = 520)
P06	caecum	flexura sin	YES	YES (inf = 2579, non-inf = 599)
P07	transversum	sigmoideum	YES	YES (inf = 1609, non-inf = 26)
P08	descendens	ascendens	YES	YES (inf = 10055, non-inf = 1118)
P09	sigmoideum	caecum	NO	YES (inf = 4519, non-inf = 2831)
P10	descendens	caecum	NO	YES (inf = 4979, non-inf = 4480)
P11	caecum	flexura sin	NO	YES (inf = 16215, non-inf = 1836)
P12	descendens	ascendens	NO	YES (inf = 8451, non-inf = 3470)
P13	ascendens	sigmoideum	NO	YES (inf = 11630, non-inf = 3597)
P14	transversum	flexura dx	NO	YES (inf = 4647, non-inf = 999)
P15	ascendens	descendens	NO	NO (inf = 436, non-inf = 595)
P16	sigmoideum	ascendens	NO	NO (inf = 2125, non-inf = 2184)

c **d**

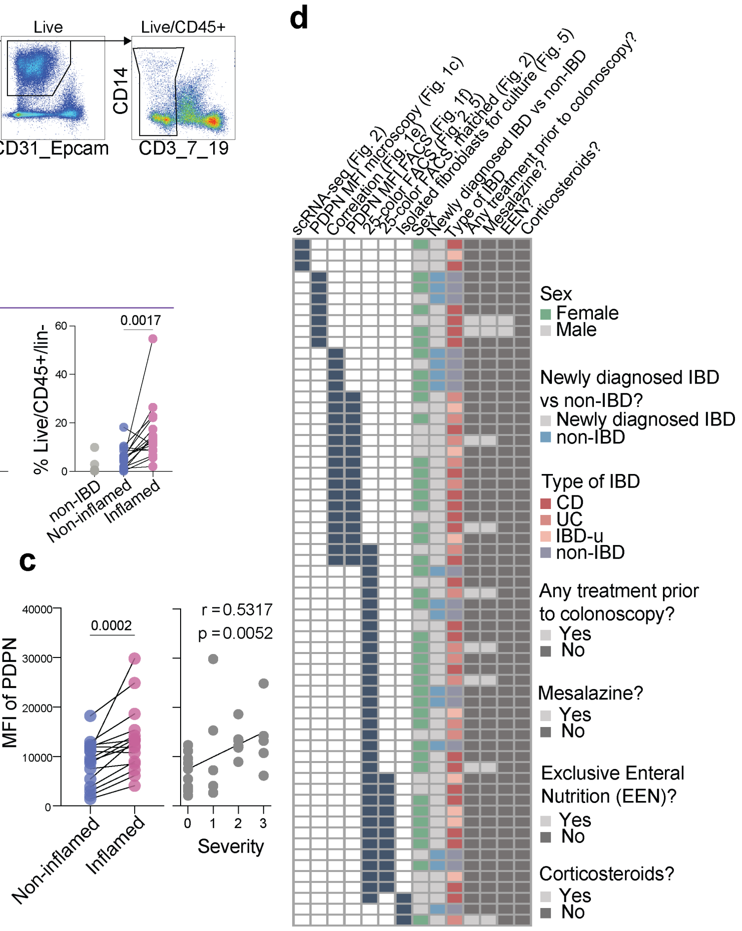

Supplementary Fig. 1 | Evaluation of inflammation in matched intestinal biopsies.

a, Gating strategy for intestinal neutrophils and eosinophils (upper); total neutrophil count and frequencies in matched non-inflamed and inflamed samples from 16 pediatric patients (lower) based on endoscopic assessment, and six non-IBD samples shown for a reference. **b**, Quality control measures for confirmation of non-inflamed and inflamed areas based on macroscopic definition made during the colonoscopy, microscopic definition established after histopathology review, and neutrophil counts, in each individual P01-P16. **c**, PDPN in $PDPN^{+}$ stroma from macroscopically (endoscopically) non-inflamed and inflamed matched mucosal colon tissue in 16 newly diagnosed IBD patients (left); correlation between PDPN levels and endoscopic severity in 26 biologically independent samples from newly diagnosed IBD patients, according to the Swedish Inflammatory Bowel Disease Register protocol adapted from the Mayo score (see “Patients, samples, and disease scoring” in Methods); samples with score 0 that after histopathology review could not be confirmed as non-inflamed were excluded from the analysis. **d**, Description of newly diagnosed pediatric patients included and grouped based on the methods performed. Wilcoxon test (two-tailed p-value) was used for matched data (**a**, **c**), and Spearman test (two-tailed p-value) was used to assess correlation (**c**).

Supplementary Fig. 2

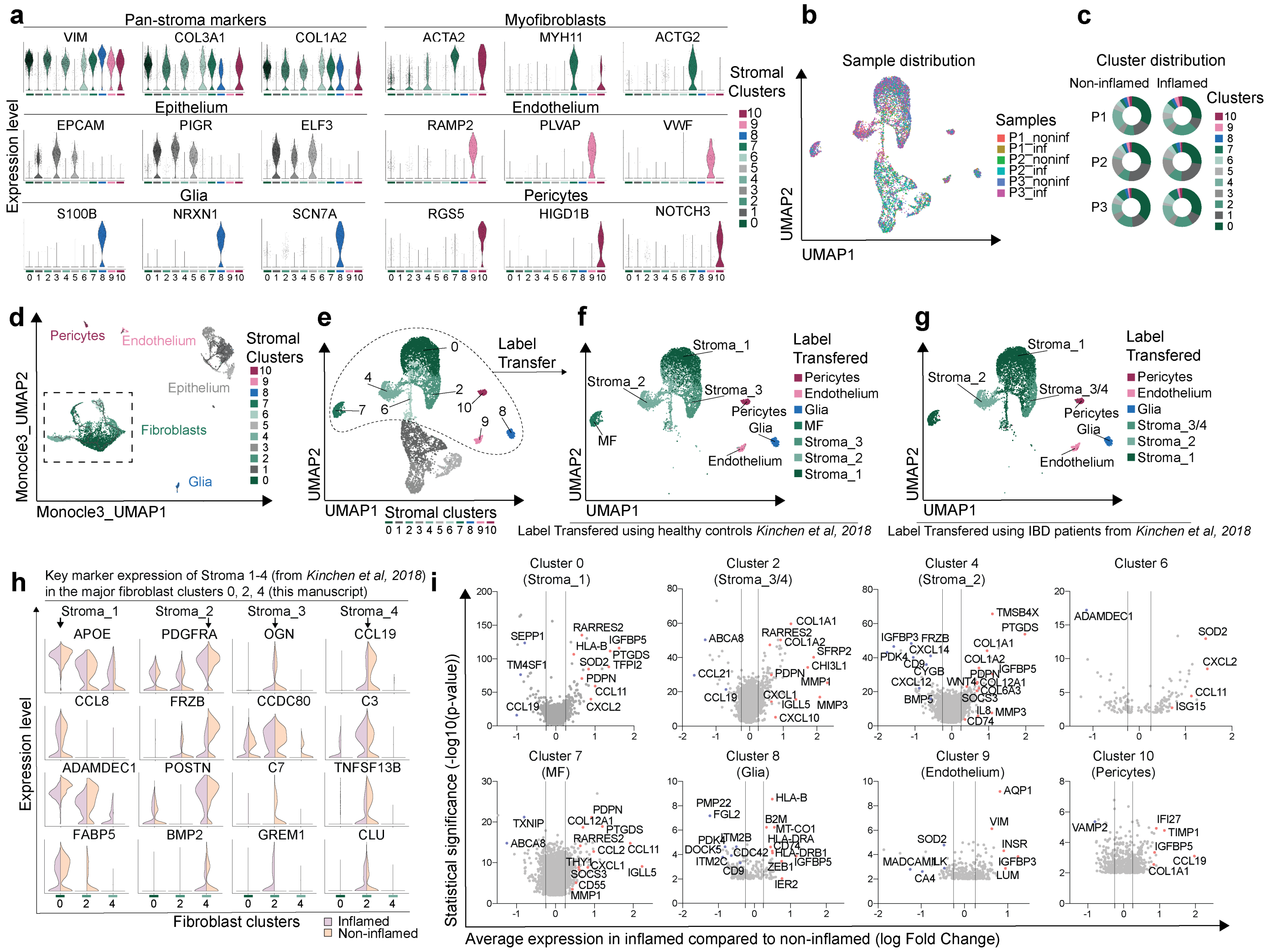

Supplementary Fig. 2 | Inflammation related changes in intestinal pediatric IBD stroma revealed by scRNA-seq. a, Differentially expressed key genes in stromal and epithelial clusters 0-10. **b**, Sample distribution in the UMAP space, non-inflamed and inflamed samples in patients P1-P3. **c**, Cluster distribution in non-inflamed and inflamed samples in patients P1-P3. **d**, Developmental trajectories among clusters 0-10 presented in Monocle 3 UMAP space. **e-g**, Stromal clusters (**e**) subjected to Label Transfer (see Methods) either using healthy controls (**f**) or IBD patients (**g**) from Kinchen et al. as a reference. **h**, Key genes for Stroma 1-4 (from Kinchen et al), differentially expressed in stroma clusters 0, 2, and 4 (this study), corresponding to the Kinchen et al definition of Stroma_1, Stroma_3/4, and Stroma_2, respectively, presented as half violin plots for inflamed (violet) and non-inflamed (orange) samples. **i**, Volcano plots illustrating differentially expressed genes in inflamed and non-inflamed tissue per cluster basis (assessed using "bimod" test, $p<0.05$); with significance depicted on y axis and average expression in inflamed compared to non-inflamed on x axis; thresholds cutting x axis are depicted at +/- 0.25.

Supplementary Fig. 3

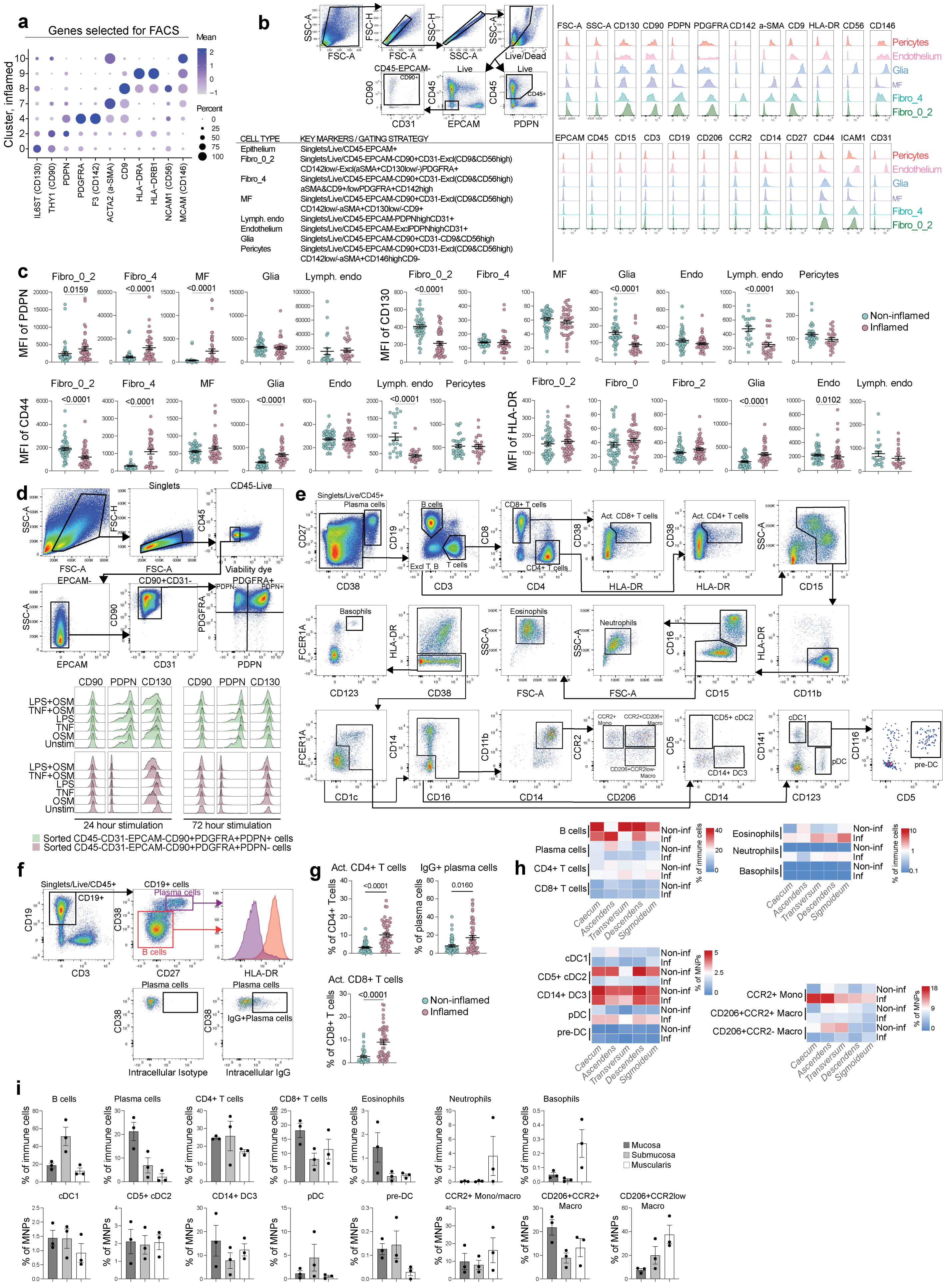

Supplementary Fig. 3 | Composition of intestinal stroma and immune cells validated across colon segments and intestinal layers using 25-color flow cytometry. a, Dot plot of gene expression, selected based on differential expression for validation on protein level, across stroma clusters; inflamed samples. **b**, Summary of FACS gating strategy used in Fig. 2g and Supplementary Fig. 3e, 3f (left), histograms of marker expression across gated subsets (right). **c**, Stroma activation markers PDPN, IL6ST (CD130), CD44, and HLA-DR across stromal subsets in 48 to 97 biologically independent non-inflamed and inflamed biopsies from children undergoing first diagnostic colonoscopy. **d**, Sorting gating strategy for cultured intestinal fibroblasts (upper panels) and levels of stroma activation markers PDPN, IL6ST (CD130) in isolated, sorted fibroblasts after 24 h and 72 h of stimulation (lower panels). **e**, Gating strategy for immune cells in *ex vivo* intestinal samples, see (**b**) for gating strategy of initial gates. **f**, Gating strategy for intracellular staining for identification of IgG^+ plasma cells in intestinal samples, see (**b**) for gating strategy of initial gates. **g**, Quantification of activated T cell frequencies and IgG^+ plasma cell frequencies in 94 to 97 biologically independent non-inflamed and inflamed biopsies from children undergoing first diagnostic colonoscopy. **h**, Heatmap illustrating percentage of total immune cells or total mononuclear phagocytes (MNP) cells across the different segments of colon in matched samples from treatment naïve pediatric patients with no inflammation in the colon (Non-inf) or total colitis (Inf). **i**, Percentage of total immune cells across dissected intestinal layers from adult IBD patients undergoing gut resection. Mean with SE in (**c**, **g**, **i**); Mann-Whitney test (two-tailed p value) was used to compare differences between two groups (**c**, **g**).

Supplementary Fig. 4

a

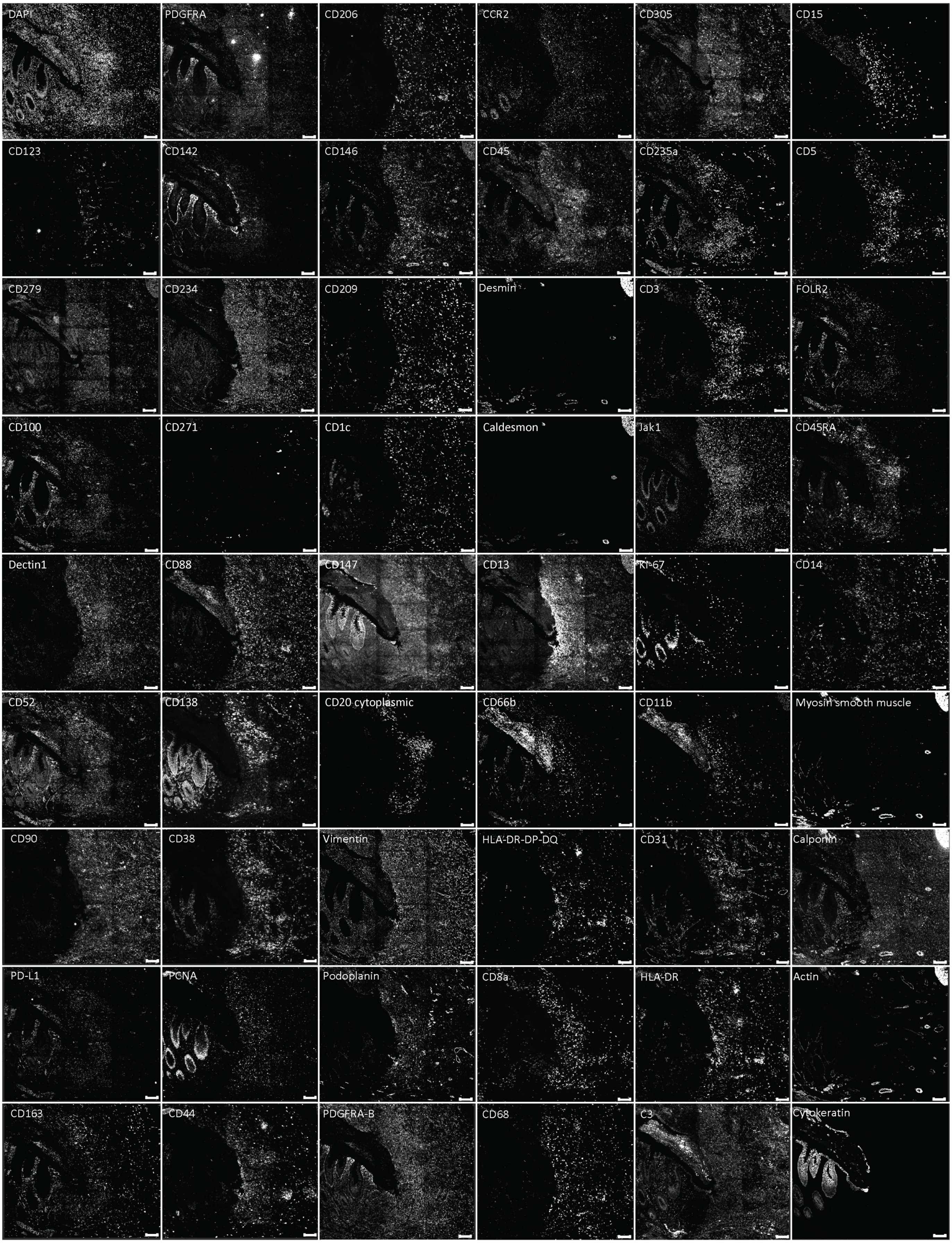

b

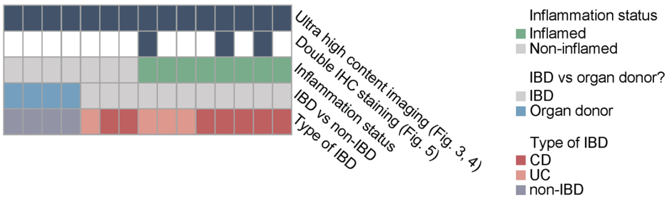

Supplementary Fig. 4 | Ultra-high content imaging using the MACSima™ imaging platform. **a**, Generation of high-dimensional fluorescence microscopy images using sequential labeling, scale bars indicate 100 µm. **b**, Description of patient characteristics, gut resection material from adult IBD patients and organ donors.

Supplementary Fig. 5

a

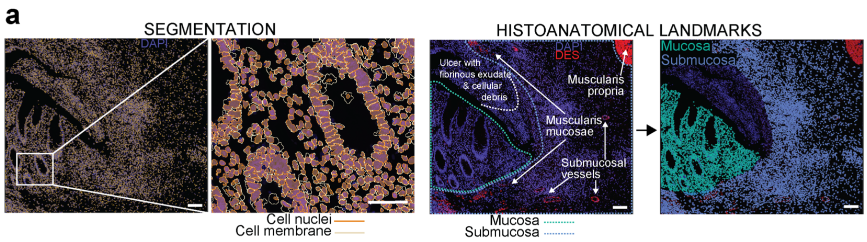

b

CELL TYPE	KEY MARKERS / GATING STRATEGY
Epithelium	Cyto+
T cells & subsets	Cyto-CD3+, Cyto-CD3+CD8+, Cyto-CD3+CD8-, Cyto-CD3+CD8+CD38+, Cyto-CD3+CD8-CD38+
B cells	Cyto-CD3-CD45RA+CD20+
Monocytes	Cyto-CD3-CD20-CD15-HLA-DR+CD88+CD14+/ CD68+CD206-/lowCD163-/low
Macrophages	Cyto-CD3-CD20-CD15-HLA-DR+CD88+CD14+/CD68+CD206+CD163+
Granulocytes	Cyto-CD3-CD20-CD14-CD68-CD15+CD66b+
Plasma cells	Cyto-CD3-CD20-CD14-CD68-CD38+CD138+
Fibro_0_2	Cyto-CD3-CD20-CD14-CD68-CD38-CD138-CD31-DES-CD15-CD142-/ lowPDGFRAB+PDGFRA+
Fibro_4	VIM+PDGFRAB++CD142++
MF/muscle cells	VIM+aSMA+DES-/low MF and VIM-/lowaSMA+DES+ muscle cells
Lymph. endo	CD271-PDPN++CD31+
Endothelium	CD31+Actin+
Glia	CD271+CD146+/low
Pericytes	CD146+aSMA+CD68-CCD31-

c BACKGATING of granulocytes, B cells, plasma cells, monocytes, macrophages, epithelium, CD8+ T cells, CD8- T cells

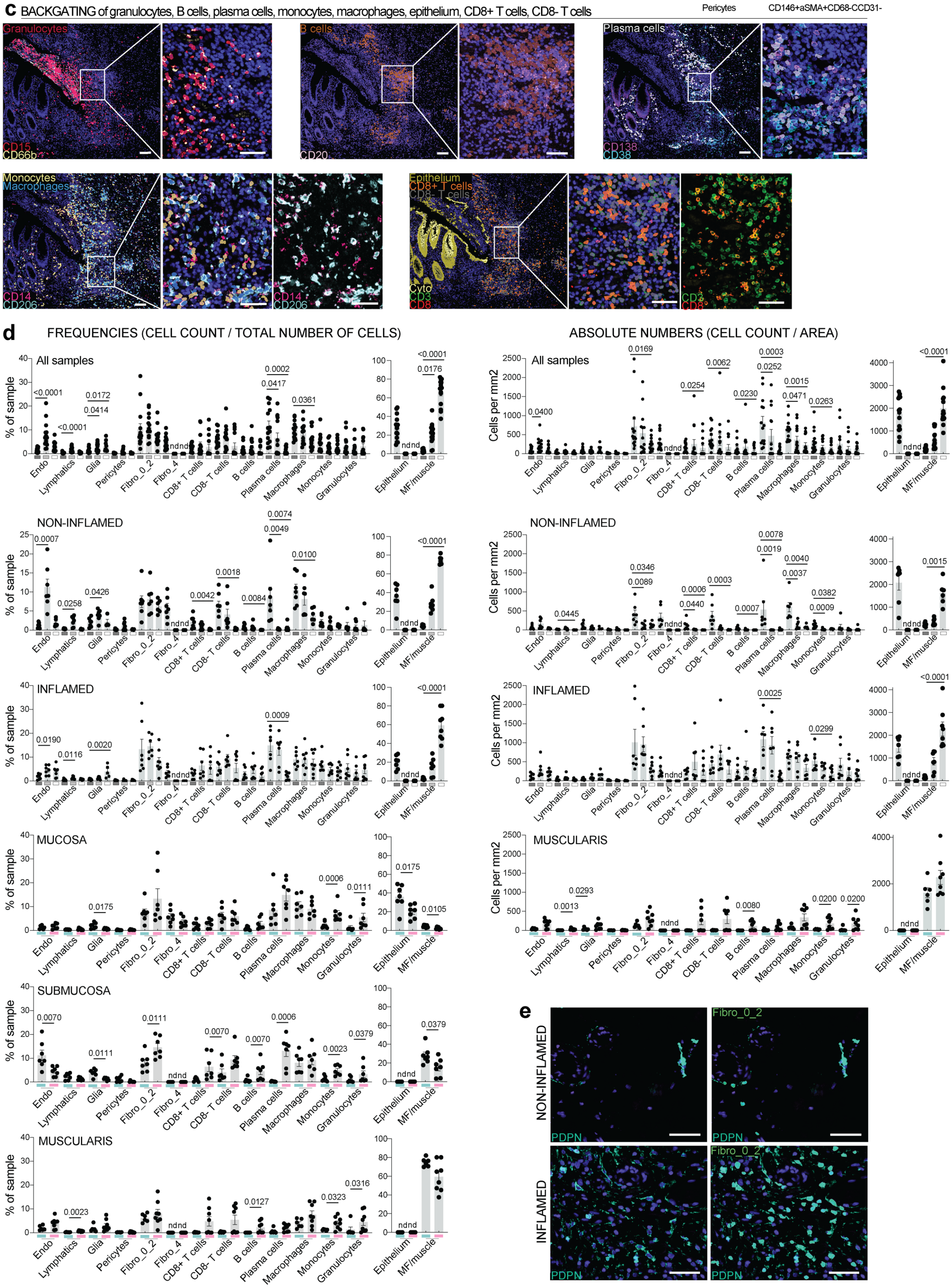

Supplementary Fig. 5 | Ultra-high content imaging reveals spatial composition of intestinal stroma and immune cells. a, Segmentation and definition of histoanatomical landmarks. **b**, Summary of gating strategy for image analyses, gating strategy is shown in Fig. 3b. **c**, Immune cell subsets backgated to the image; scale bars indicate 100 µm in images on the left and 50 µm in images presented in higher magnification. **d**, Cell frequencies (percentage per sample) and absolute numbers (cells/mm^2) of cell subsets quantified across intestinal layers in non-inflamed and inflamed samples from adult gut resections; lymphoid tissue was excluded from the analyses. **e**, Representative images of non-inflamed and inflamed submucosa showing Fibro_0_2 (right) population and overlayed with PDPN staining, scale bars indicate 50 µm. Mean with SE in (**d**); Mann-Whitney test (two-tailed p value) was used to compare differences between two non-inflamed and inflamed samples (**d**), and differences between the layers were assessed using Kruskal-Wallis test with Dunn's multiple comparisons test to compare differences between mucosa vs submucosa and mucosa vs muscularis (**d**).

Supplementary Fig. 6

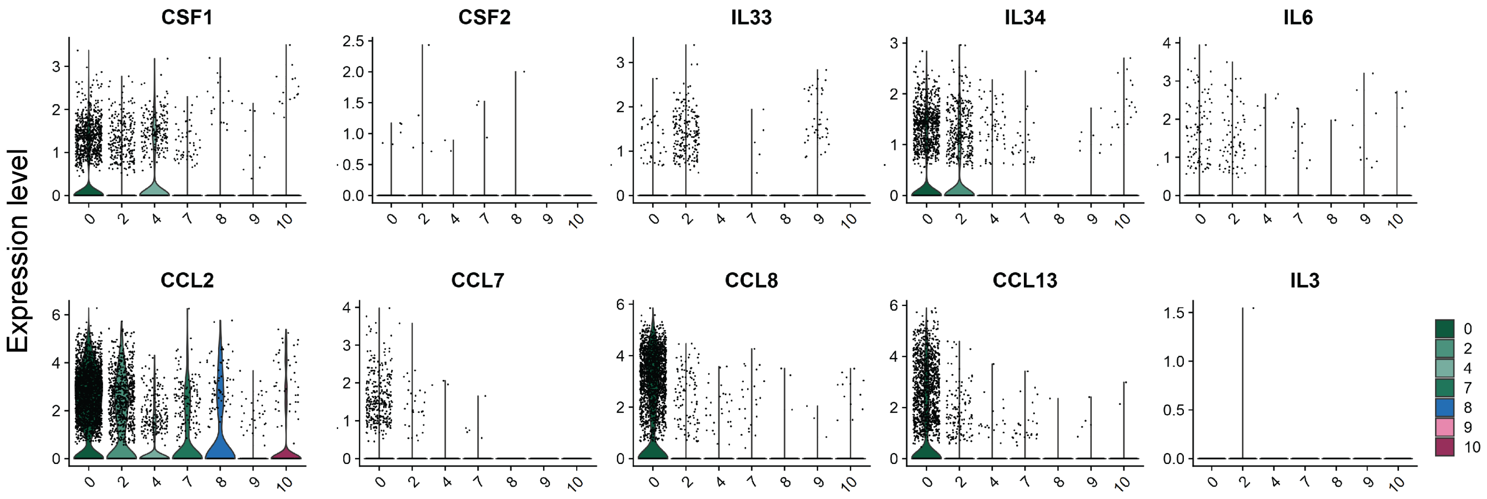

Supplementary Fig. 6 | Individual gene expression from monocyte/macrophage differentiation/recruitment score. Expression level of indicated cytokines in major stromal clusters from our scRNA-seq: fibroblasts in clusters 0, 2, 4 (Fibro_0, Fibro_2, Fibro_4), myofibroblasts in cluster 7, glia in cluster 8, endothelium in cluster 9, and pericytes in cluster 10.

Supplementary Fig. 7

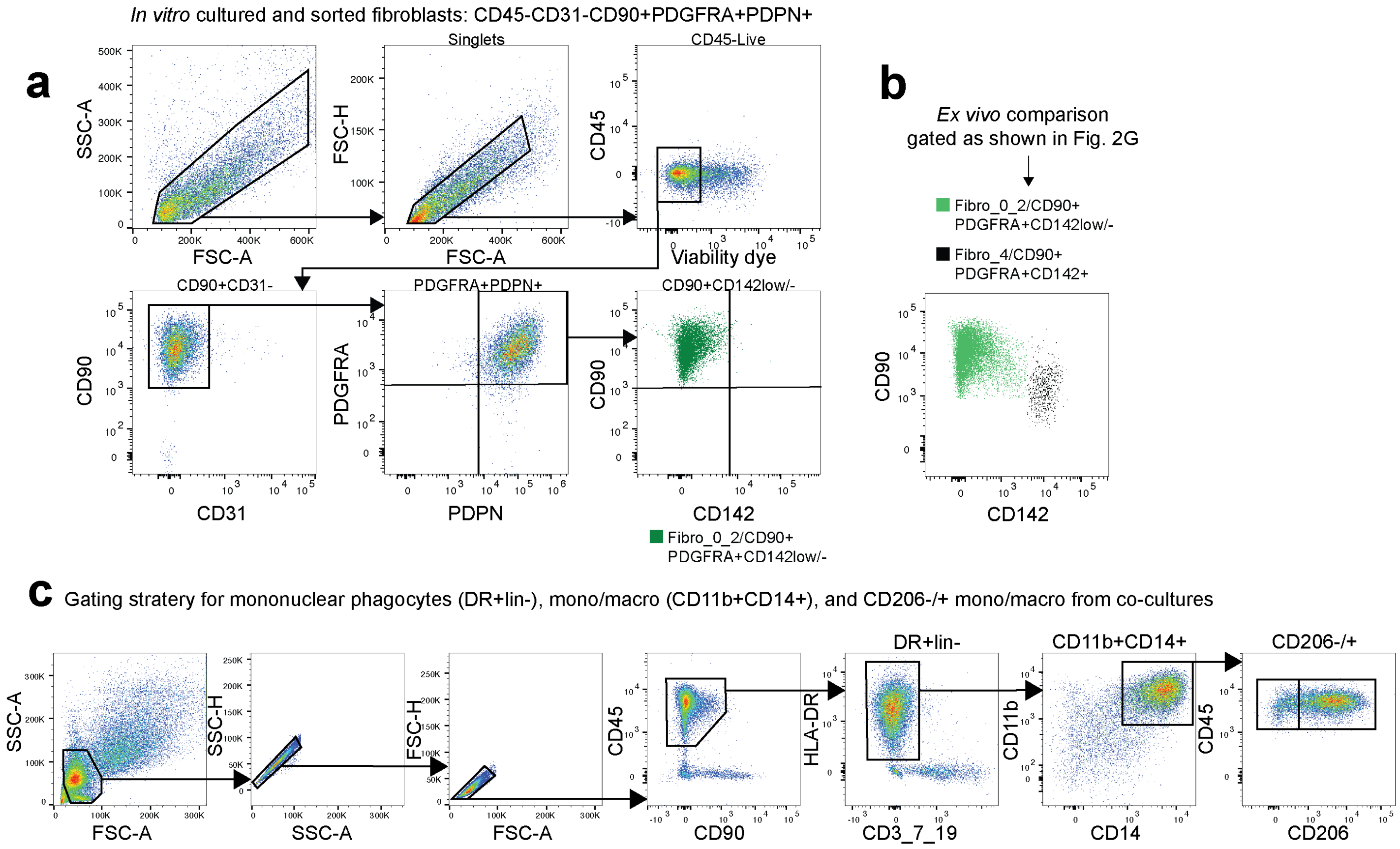

Supplementary Fig. 7 | PDGFRA+CD142low/- fibroblasts in vitro. **a**, Gating strategy and phenotype of fibroblasts used for co-culture experiments with monocytes to generate data in Fig. 5 and Supplementary Fig. 8, and for monocultures of fibroblasts used for data in Fig. 5i, and Supplementary Fig. 8f. **b**, *Ex vivo* data of fibroblast subsets Fibro_0_2 and Fibro_4. **c**, Gating strategy for cultured DR^{+}lin^{-} mononuclear phagocytes used for data in Fig. 5b-e, 5g, 5h, 5k, 5l and Supplementary Fig. 8a, 8c, 8d, 8i; monocytes/macrophages (CD11b^{+}CD14^{+}) used for data in Fig. 5f, 5j and Supplementary Fig. 8e; and CD206$^{-/+}$ monocytes/macrophages used for data in Supplementary Fig. 8g.

Supplementary Fig. 8

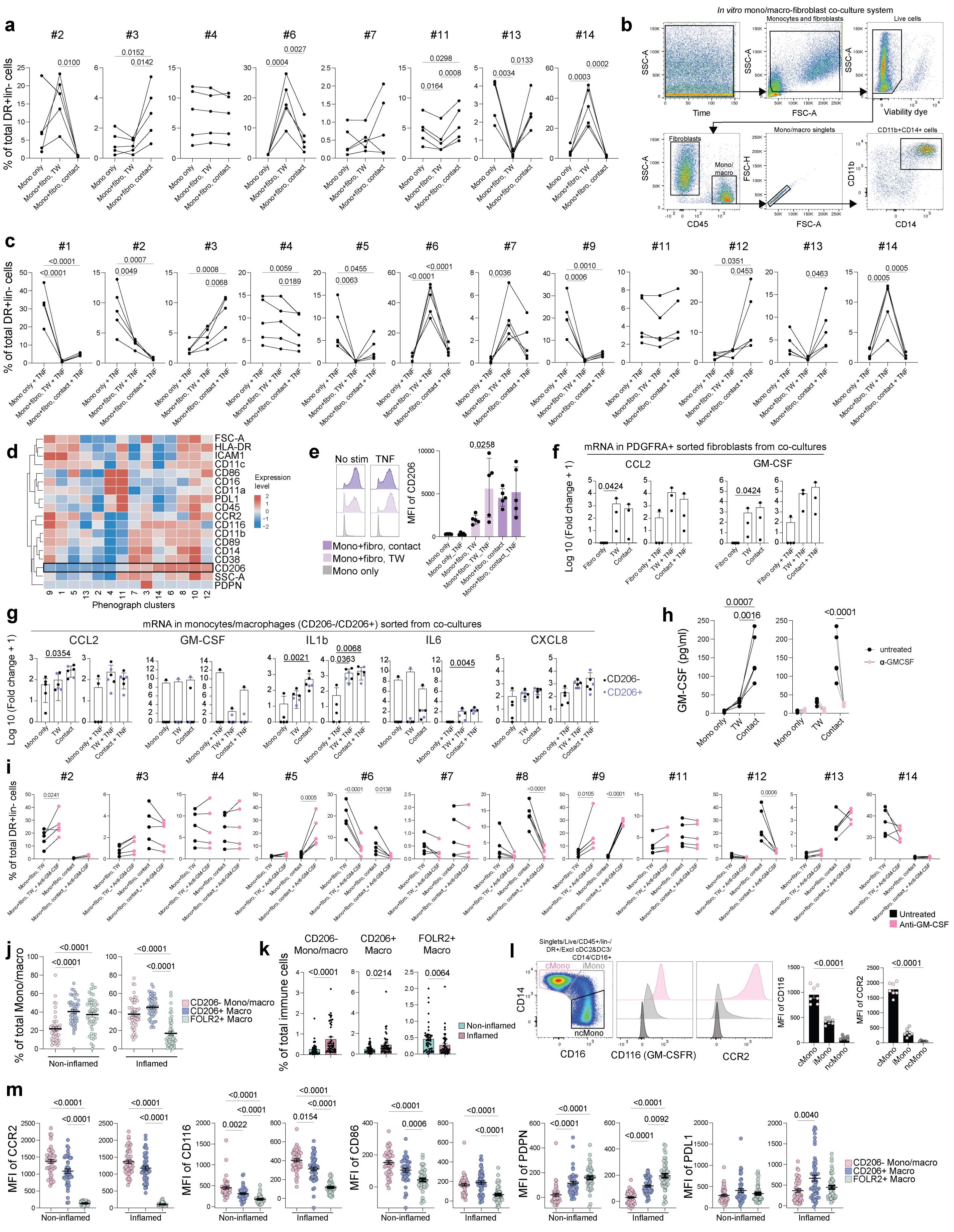

Supplementary Fig. 8 | PDGFRA^{+} fibroblasts guide monocyte to macrophage transition through GM-CSF. **a**, Quantification of cell frequencies across culture conditions: monocytes only, monocytes separated from fibroblasts in transwells (TW), and monocytes cultured in contact with fibroblast. **b**, Overview of monocyte-fibroblast co-culture system. **c**, Quantification of cell frequencies across conditions in Phenograph clusters, with TNF stimulation. **d**, Heatmap of marker expression across clusters, with TNF stimulation. **e**, Expression of macrophage marker CD206 across conditions in total monocytes/macrophages with and without TNF stimulation; same data is shown in Fig. 5f, but without TNF stimulation. **f**, qPCR on fibroblasts sorted from the cultures with or without monocytes in different culture conditions, with or without TNF stimulation. **g**, qPCR on monocytes/macrophages sorted from the cultures with or without fibroblasts in different culture conditions, with or without TNF stimulation. **h**, Quantification of soluble GM-CSF in co-culture supernatants from different culture conditions, with or without anti-GM-CSF antibodies. **i**, Quantification of cell frequencies across Phenograph clusters, comparing with and without anti-GM-CSF antibodies. **j**, Frequencies of intestinal CD206^{-} monocytes/macrophages (pink), CD206^{+} macrophages (blue), and FOLR2^{+} macrophages (green). **k**, Differences in CD206^{-} Mono/Macro, CD206^{+} Macro, and FOLR2^{+} frequencies among total immune cells (CD45^{+}) in non-inflamed and inflamed pediatric mucosal biopsy samples. **l**, Quantification of GM-CSF receptor (CD116) and CCR2 in circulating classical monocytes (cMono), intermediate monocytes (iMono), and non-classical monocytes (ncMono), for details on gating strategy see Supplementary 3e. **m**, Quantification of marker expression across intestinal monocyte/macrophage subsets in non-inflamed and inflamed samples. Mean with SD in (**e**-**g**), mean with SE in (**j**-**m**); Mann-Whitney test (two-tailed p value) was used to compare differences between two non-inflamed and inflamed samples (**k**), differences among more than two groups were assessed using one-way ANOVA with Holm-Šídák's multiple comparisons test (**a**, **c**, **e**, **h** left, **i**), two-way ANOVA with Holm-Šídák's multiple comparisons test (**h** right), and Kruskal-Wallis with Dunn's multiple comparisons test (**f**, **g**, **j**, **l**, **m**).

Supplementary Fig. 9

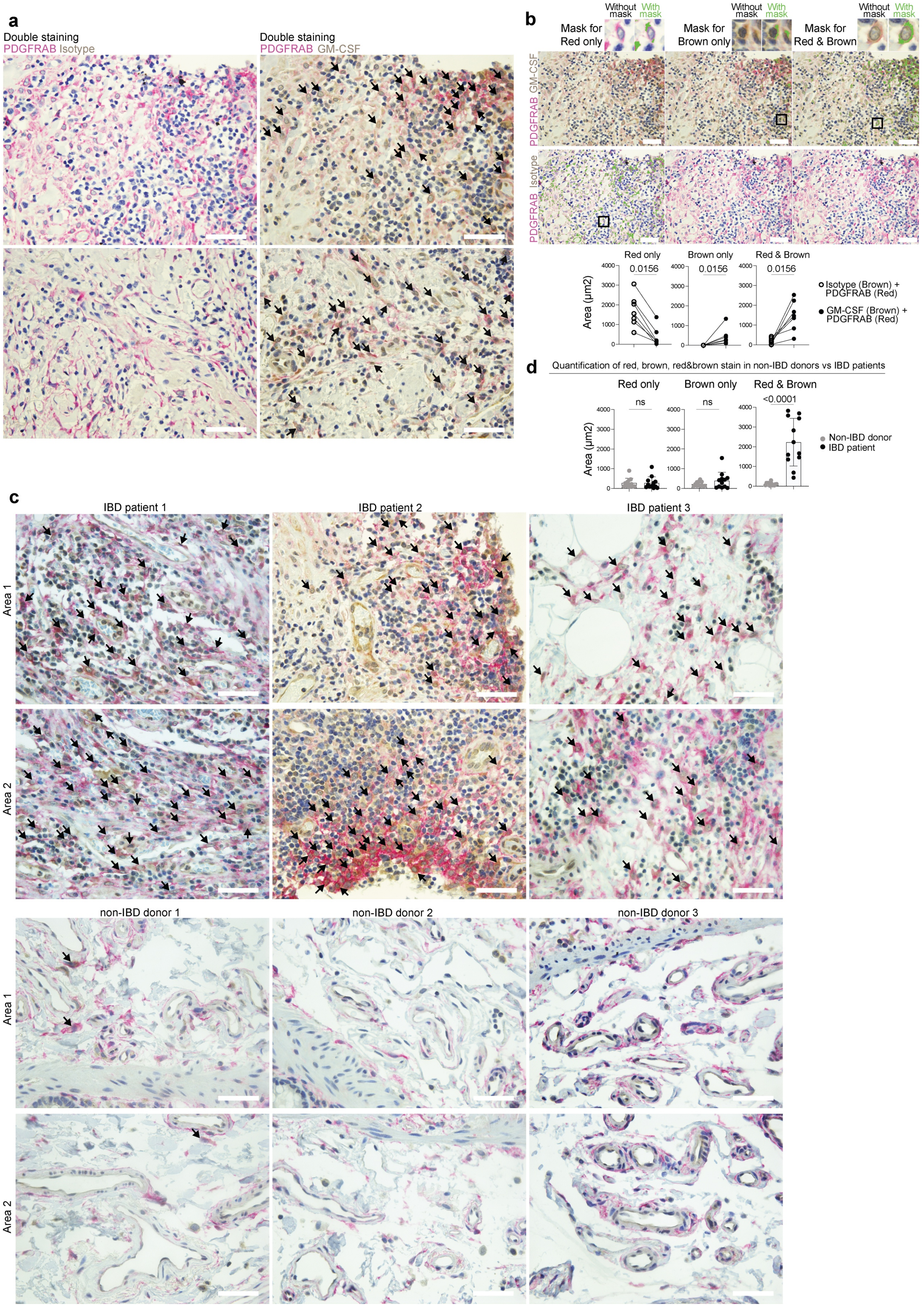

Supplementary Fig. 9 | Double staining of intestinal PDGFRAB$^+$ fibroblasts and GM-CSF. **a**, Double staining of intestinal PDGFRAB$^+$ fibroblasts (red) and either isotype control (brown, left) or GM-CSF (brown, right) in inflamed submucosa, scale bar indicates 50 µm. **b**, Illustration of analytic pipeline where PDGFRAB$^+$ fibroblasts (red) and either isotype control (brown, lower panel) or GM-CSF (brown, upper panel) were compared with respect to red only, brown only, or red and brown staining; quantification of area of red, brown, or red and brown stain in matched areas of consecutive sections is provided below the images. **c**, Double staining of intestinal PDGFRAB$^+$ fibroblasts (red) and GM-CSF (brown) in submucosa of IBD patients (upper panels) and non-IBD donors (lower panels), scale bar indicates 50 µm. **d**, Quantification of area of red, brown, or red and brown stain in submucosa of IBD patients and non-IBD donors. Black arrows indicate spindle-shaped PDGFRAB$^+$GM-CSF$^+$ cells exhibiting activated fibroblast morphology with large flat nuclei and prominent nucleoli (**a**, **c**). Wilcoxon test for matched data (two-tailed p value) (**b**) was used and Mann-Whitney test (two-tailed p value) was used to compare differences between two groups (**d**).

Supplementary Fig. 10

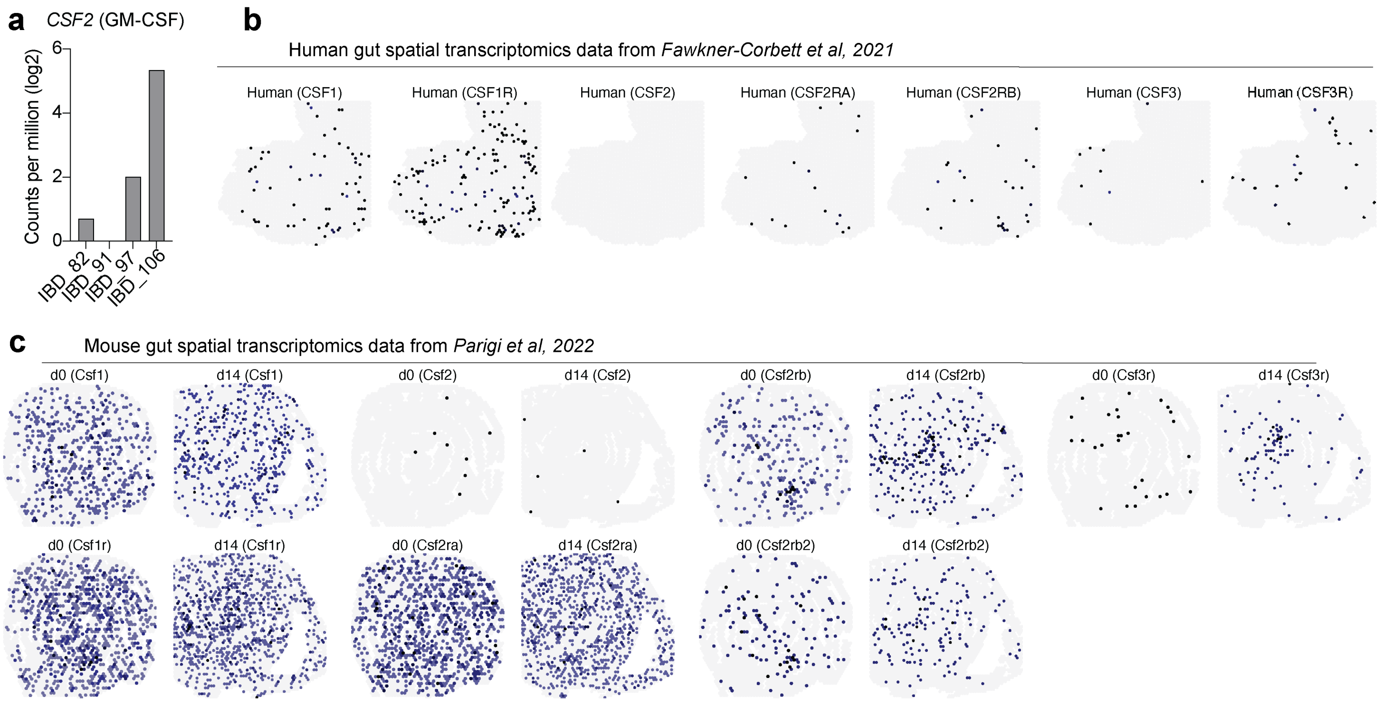

Supplementary Fig. 10 | CSF-2 (GM-CSF) transcriptional levels. a, Detection of CSF-2 (GM-CSF) mRNA in freshly *ex vivo* semi-bulk sorted intestinal fibroblasts from four IBD patients (live/CD45$^-$CD31$^-$CD90$^+$PDPN$^+$, for gating strategy see Fig. 1d). **b**, Colony stimulating factor and receptor expression in human gut spatial transcriptomics data from *Fawkner-Corbett et al, 2021*. **c**, Colony stimulating factor and receptor expression in mouse gut injury dynamics model at day 0 (d0) and during the gut healing phase (d14), spatial transcriptomics data from *Parigi et al, 2022*.

Supplementary Table 1. Clinical characteristics of all recruited patients

	IBD	CD	UC	IBD-u	non-IBD
Group size, n	48	26	13	9	17
**General details**					
Age at sampling y, median (range)	14.9 (4.2-18.2)	13.7 (4.2-18.2)	15.1 (8.0-17.9)	15.8 (10.7-17.3)	11.4 (3.1-16.0)
Age at diagnosis y, median (range)	14.6 (4.2-18.2)	13.7 (4.2-18.2)	15.1 (8.0-17.9)	14.4 (5.8-17.3)	10 (3.1-15.1)
Newly diagnosed IBD, n (%)	48 (100)	26 (100)	13 (100)	9 (100)	n/a
Sex, female/male	18/30	10/16	3/10	5/4	6/11
**Disease activity at sampling, n (%)**					
Physician's global assessment (score)*					
- Remission (0)	0 (0)	0 (0)	0 (0)	0 (0)	5 (29.4)
- Mild (1)	7 (15.6)	3 (11.5)	3 (23.1)	1 (11.1)	11 (64.7)
- Moderate (2)	33 (73.3)	18 (69.2)	8 (61.5)	7 (77.8)	1 (5.9)
- Severe (3)	5 (11.1)	3 (11.5)	2 (15.4)	0 (0)	0 (0)
Endoscopic disease severity (score)†					
- Normal or inactive (0)	4 (8.3)	4 (15.4)	0 (0)	0 (0)	17 (100)
- Mild (1)	11 (22.9)	8 (30.8)	2 (15.4)	1 (11.1)	0 (0)
- Moderate (2)	18 (37.5)	8 (30.8)	6 (46.2)	4 (44.4)	0 (0)
- Severe (3)	15 (31.3)	6 (23.1)	5 (38.5)	4 (44.4)	0 (0)
**Symptomatology**					
Months with symptoms prior to the sampling, median (range)‡	5 (1-48)	6.5 (2-48)	3 (1-7)	6 (2-12)	9 (4-24)
Symptoms, n (%)					
- Abdominal pain	35 (72.9)	22 (84.6)	8 (61.5)	5 (55.6)	7 (41.2)
- Persistent diarrhea	29 (60.4)	16 (61.5)	7 (53.8)	6 (66.7)	4 (23.5)
- Bloody stool	29 (60.4)	12 (46.2)	12 (92.3)	5 (55.6)	10 (58.8)
- Fatigue	6 (12.5)	5 (19.2)	1 (7.7)	0 (0)	0 (0)
- Unintended weight loss and/or growth problems	15 (31.3)	12 (46.2)	1 (7.7)	2 (22.5)	1 (5.9)
**Treatment details, n (%)**					
Any treatment prior§ to the sampling	9 (18.8)	4 (15.4)	5 (38.5)	0 (0)	0 (0)
- Mesalamine	9 (18.8)	4 (15.4)	5 (38.5)	0 (0)	0 (0)
- Corticosteroids	0 (0)	0 (0)	0 (0)	0 (0)	0 (0)
- 6-mercaptopurine	0 (0)	0 (0)	0 (0)	0 (0)	0 (0)
- Methotrexate	0 (0)	0 (0)	0 (0)	0 (0)	0 (0)
- anti-TNF	0 (0)	0 (0)	0 (0)	0 (0)	0 (0)
- Exclusive enteral nutrition	2 (4.2)	2 (7.7)	0 (0)	0 (0)	0 (0)
- Surgery	0 (0)	0 (0)	0 (0)	0 (0)	0 (0)

Any treatment after[¶] the sampling	40 (83.3)	20 (76.9)	12 (92.3)	8 (88.9)	0 (0)
- Mesalamine	32 (66.7)	15 (62.5)	9 (69.2)	5 (55.6)	0 (0)
- Corticosteroids	21 (43.8)	9 (37.5)	7 (53.8)	1 (11.1)	0 (0)
- 6-mercaptopurine	6 (12.5)	5 (20.8)	0 (0)	0 (0)	0 (0)
- Methotrexate	3 (6.3)	3 (12.5)	0 (0)	0 (0)	0 (0)
- anti-TNF	15 (31.3)	9 (37.5)	5 (38.5)	1 (11.1)	0 (0)
- Exclusive enteral nutrition	8 (16.7)	7 (29.2)	0 (0)	1 (11.1)	0 (0)
- Surgery	1 (2.1)	1 (4.2)	0 (0)	0 (0)	0 (0)

n/a: not applicable; TNF: tumor necrosis factor; y: years

[*]Physician's global assessment (score) (Hyams et al, J. Pediatr. Gastroenterol. Nutr., 2015)

[†]Adapted from Mayo endoscopy index (Schroeder et al, N. Engl. J. Med., 1987)

[‡]Patients undergoing first diagnostic colonoscopy only

[§]Ongoing and ever received

[¶]Until the last follow-up

Supplementary Table 2. Individual patient characteristics and laboratory data of the newly diagnosed patient cohort (Fig. 1)

Disease type	Disease activity PGA*/Endoscopy†	Calprotectin, mg/kg	Hemoglobin, g/L	Albumin, g/L	ESR, mm/hour	CRP, ng/L	Platelets, $x10^9/L$
**UC**	2/1	365	138	44	1	<1	162
**IBD-u**	2/3	4450	128	41	7	<1	176
**UC**	2/3	466	151	44	1	<1	202
**CD**	2/1	768	119	40	5	5	306
**UC‡**	2/2	3690	131	38	21	<1	405
**IBD-u**	2/2	1980	122	41	8	<1	239
**UC**	1/2	398	112	44	6	1	364
**CD**	3/3	3550	109	19	65	43	545
**UC**	1/2	1470	136	44	7	<1	392
**CD**	2/1	2030	117	37	14	8	299
**CD**	2/0	1760	124	40	9	<1	281
**CD**	2/1	616	119	40	21	5	410
**CD**	3/3	1580	100	32	75	80	474
**UC**	2/3	1993	127	44	14	<1	391
**CD**	2/2	438	124	27	28	15	305
**IBD-u**	1/2	2130	144	43	14	5	250

ESR: Erythrocyte sedimentation rate; CRP: C-reactive protein; PGA: Physician's global assessment

*Physician's global assessment (Hyams et al, J. Pediatr. Gastroenterol. Nutr., 2015): 0=remission, 1=mild, 2=moderate, 3=severe.

†Endoscopy score adapted from Mayo endoscopy index (Schroeder et al, N. Engl. J. Med., 1987): 0=normal or inactive, 1=mild, 2=moderate, 3=severe. If endoscopic severity varied in the colon, the maximal endoscopy score is indicated.

‡UC atypical, according to the Diagnostic Criteria in Children, as described in Birimberg-Schwartz et al, 2017 (doi:10.1093/ecco-jcc/jjx053)

Supplementary Table 3: Antibodies

Antibody	Fluorochrome	Clone	Company	Application/dilution
Anti-PDPN	Unconjugated	NZ-1	OriGene	IF/1:100, Clarity/1:10
Anti-αSMA	Unconjugated	1A4	Abcam	Clarity/1:50
Goat Anti-Rat IgG	AF488	-	Thermo Fisher	IF/1:400, Clarity/1:100
Goat Anti-Mouse IgG2a	AF594	-	Thermo Fisher	Clarity/1:100
Anti-ICAM1	BV421	HA58	BD	FACS/1:50
Dead cell stain	DCM aqua	-	Thermo Fisher	FACS/1:100
Anti-PDL1	BV605	29E.2A3	Biolegend	FACS/1:50
Anti-CD11a	BV650	HI111	BD	FACS/1:100
Anti-CD11b	BV711	ICRF44	Biolegend	FACS/1:100
Anti-CD3	BV750	SK7	Biolegend	FACS/1:50
Anti-CD7	BV750	M-T701	BD	FACS/1:50
Anti-CD19	BV750	HIB19	BD	FACS/1:50
Anti-HLA-DR	BV786	L243	Biolegend	FACS/1:50
Anti-CD206	BB515	19.2	BD	FACS/1:50
Anti-CD1c	BB700	F10/21A3	BD	FACS/1:50
Anti-CD31	PE-CF594	WM59	Biolegend	FACS/1:100
Anti-CD31	PB	WM59	Biolegend	FACS/1:50
Anti-EPCAM	PE-CF594	9C4	Biolegend	FACS/1:800
Anti-CD123	PE-CY5	9F5	BD	FACS/1:100
Anti-CD11c	PE-Cy5.5	BU15	Thermo Fisher	FACS/1:200
Anti-PDPN	PE-Cy7	NC-08	Biolegend	FACS/1:100
Anti-CLEC9A	AF647	3A4	BD	FACS/1:50
Anti-CD45	Alexa700	HI30	BioLegend	FACS/1:100
Anti-CD45	PE/CF594	HI30	BD	FACS/1:50
Anti-CD14	APC-Cy7	M5E2	Biolegend	FACS/1:200
Anti-CD14	BV570	M5E2	Biolegend	FACS/1:100
Anti-CD90	BUV395	5E10	BD	FACS/1:400
Anti-CD90	BV605	5E10	BD	FACS/1:50
Anti-CCR2	BUV615-P	LS132.1D9	BD	FACS/1:100
Anti-CD15	BUV661	W6D3	BD	FACS/1:400
Anti-CD16	BUV737	3G8	BD	FACS/1:400
Anti-CD16	BV785	3G8	Biolegend	FACS/1:100
Anti-CD86	BUV805	2A9-1	BD	FACS/1:25

Anti-Vimentin	AF488	RV202	BD	FACS/1:50
Anti-CD19	PE-Cy5.5	J3-119	Beckman	FACS/1:100
Anti-IgG	BUV563	G18-145	BD	FACS/1:200
Isotype control	BUV563	X40	BD	FACS/1:200
Anti-aSMA	AF488	1A4	R&D	FACS/1:200
Anti-CD44	BV570	IM7	Biolegend	FACS/1:100
Anti-CD206	BV605	19.2	BD	FACS/1:50
Anti-PDGFRA	BV650	αR1	BD	FACS/1:25
Anti-HLA-DR	BV711	L243	Biolegend	FACS/1:100
Anti-CD27	BV750	O323	BD	FACS/1:100
Anti-CD130	BB700	AM64	BD	FACS/1:100
Anti-CD146	PE	P1H12	Biolegend	FACS/1:400
Anti-CD15	APC	W6D3	Biolegend	FACS/1:100
Anti-CD56	BUV496	NCAM16.2	BD	FACS/1:100
Anti-CD38	BUV661	G46-6	BD	FACS/1:400
Anti-CD142	BUV737	HTF-1	BD	FACS/1:100
Anti-CD142	PE	HTF-1	LSBio	FACS/1:50
Anti-CD9	BUV805	M-L13	BD	FACS/1:25
Anti-CD38	BV421	HIT2	BD	FACS/1:50
Anti-CD3	BV570	UCHT1	Biolegend	FACS/1:25
Anti-CD141	BV711	1A4	BD	FACS/1:25
Anti-CD116	PE	4H1	Biolegend	FACS/1:50
Anti-PDL1	PE-CF594	29E.2A3	Biolegend	FACS/1:400
Anti-CD4	PE-CY5	OKT4	Biolegend	FACS/1:200
Anti-FOLR2	APC	94b/FOLR2	Biolegend	FACS/1:200
Anti-CD123	BUV395	7G3	BD	FACS/1:100
Anti-CD8	BUV496	RPA-T8	BD	FACS/1:25
Anti-CD5	BUV563	UCHT2	BD	FACS/1:50
Anti-CD15	BUV661	W6D3	BD	FACS/1:400

IF, immunofluorescence; FACS, flow cytometry.

Supplementary Table 4: Antibodies for ultra-high content imaging

Species	Antigen	Clone	Dilution	Supplier	Order number	Dye
human	CD206	EPR22489-7	200	Abcam	ab254471	-
human	PDGFRA	-	400	Novus Biologicals	AF-307-SP	-
human	CCR2	K036C2	50	Biolegend	357207	APC
human	C3	EPR2988	200	Abcam	ab196639	APC
human	PD-L1	28-8	25	Abcam	ab224027	FITC
human	FOLR2	OTI4G6	100			FITC
human	CD163	EDHu-1	50	Novus Biologicals	NB110-40686F	FITC
human, mouse, rat	PDGFRA_B	Y92	50	Abcam	ab196376	FITC
human	CD142_F3	HTF-1	50	LSBio	LS-C751060	PE
human	CD146	EPR3208	100	Biolegend	361006	PE
human	CD123	REA918	10	Miltenyi Biotec	130-115-265	APC
human	CD45	5B1	13	Miltenyi Biotec	130-113-114	APC
human	CD209 (DC-SIGN)	REAL690	50	Miltenyi Biotec	130-125-079	APC
human	CD15	VIMC6	50	Miltenyi Biotec	130-113-482	APC
human	CD235a	REA175	50	Miltenyi Biotec	130-118-356	APC
human	CD3	REA1151	50	Miltenyi Biotec	130-120-269	APC
human	CD44	DB105	50	Miltenyi Biotec	130-113-331	APC
human	CD88 (C5AR)	REA1213	50	Miltenyi Biotec	130-123-380	APC
human	CD20 Cytoplasmic	REA543	11	Miltenyi Biotec	130-108-290	APC
human	CD8a	REA1024	50	Miltenyi Biotec	130-117-202	APC

human	CD100	REA316	25	Miltenyi Biotec	130-104-600	FITC
human	CD1c (BDCA-1)	REAL1005	50	Miltenyi Biotec	130-127-024	FITC
human	CD147	REA282	50	Miltenyi Biotec	130-124-221	FITC
human	CD52	REA164	50	Miltenyi Biotec	130-123-680	FITC
human	CD66b	REA306	50	Miltenyi Biotec	130-123-694	FITC
human	CD45RA	REA562	50	Miltenyi Biotec	130-113-365	FITC
human, mouse	Ki-67	REA183	50	Miltenyi Biotec	130-117-691	FITC
human	HLA-DR	REAL550	50	Miltenyi Biotec	130-123-076	FITC
human	Myosin Smooth Muscle	REA1107	50	Miltenyi Biotec	130-119-313	FITC
human	Vimentin	REA409	50	Miltenyi Biotec	130-116-508	FITC
human	Cytokeratin	REA831	50	Miltenyi Biotec	130-112-743	FITC
human	Actin (Smooth Muscle)	REAL650	100	Miltenyi Biotec	130-123-363	FITC
human	CD279 (PD1)	REA1165	10	Miltenyi Biotec	130-120-382	PE
human	CD234 (DARC)	REA376	100	Miltenyi Biotec	130-125-845	PE
human	CD305 (LAIR-1)	REA447	50	Miltenyi Biotec	130-126-091	PE
human	CD90	REAL677	50	Miltenyi Biotec	130-124-176	PE

human	CD38	REAL719	50	Miltenyi Biotec	130-126-438	PE
human	CD5	REAL760	50	Miltenyi Biotec	130-125-077	PE
human, mouse, rat	PCNA	REA858	50	Miltenyi Biotec	130-114-512	PE
human	Dectin-1	REA515	50	Miltenyi Biotec	130-121-993	PE
human	CD271 (LNGFR)	REAL709	13	Miltenyi Biotec	130-125-053	PE
human	Caldesmon	REA1120	50	Miltenyi Biotec	130-119-344	PE
human	CD13	REAL771	50	Miltenyi Biotec	130-125-787	PE
human	CD138	REA929	50	Miltenyi Biotec	130-115-479	PE
human, mouse, rat	Jak1	REA700	50	Miltenyi Biotec	130-110-549	PE
human	HLA-DR, DP, DQ	REA332	50	Miltenyi Biotec	130-120-715	PE
human	CD31	REA1312	50	Miltenyi Biotec	120-060-718	PE
human	CD14	REA1314	50	Miltenyi Biotec	120-060-722	PE
human	CD11b	REA1321	50	Miltenyi Biotec	120-060-726	PE
human	CD68	REA1306	50	Miltenyi Biotec	120-060-257	PE
human, rat	Calponin	REA1104	50	Miltenyi Biotec	130-119-071	PE
human	Podoplanin	REA446	50	Miltenyi Biotec	130-117-687	PE
human	Desmin	REA1134	50	Miltenyi Biotec	130-119-490	PE
donkey	Anti-goat	-	100	Invitrogen	A16006	FITC

donkey	Anti-rabbit	-	50	Invitrogen	12-4739-81	PE
rat	Anti-mouse	X-56	50	Miltenyi Biotec	130-119-585	PE
