# Supplementary material for: Intestinal stroma guides monocyte differentiation to macrophages through GM-CSF
Source: Nat Commun. 2024 Feb 26;15:1752. doi: 10.1038/s41467-024-46076-3 (PMC10897309; doi:10.1038/s41467-024-46076-3)
Supplement: Supplementary file 3 — Reporting Summary [file 41467_2024_46076_MOESM3_ESM.pdf]

nature portfolio

Corresponding author(s): Egle Kvedaraite
Mattias Svensson

Last updated by author(s): Jan 28, 2024

# Reporting Summary

Nature Portfolio wishes to improve the reproducibility of the work that we publish. This form provides structure for consistency and transparency in reporting. For further information on Nature Portfolio policies, see our Editorial Policies and the Editorial Policy Checklist.

## Statistics

For all statistical analyses, confirm that the following items are present in the figure legend, table legend, main text, or Methods section.

| n/a | Confirmed | |
|---|---|---|
| ☐ | ☒ | The exact sample size (*n*) for each experimental group/condition, given as a discrete number and unit of measurement |
| ☐ | ☒ | A statement on whether measurements were taken from distinct samples or whether the same sample was measured repeatedly |
| ☐ | ☒ | The statistical test(s) used AND whether they are one- or two-sided<br>*Only common tests should be described solely by name; describe more complex techniques in the Methods section.* |
| ☐ | ☒ | A description of all covariates tested |
| ☐ | ☒ | A description of any assumptions or corrections, such as tests of normality and adjustment for multiple comparisons |
| ☐ | ☒ | A full description of the statistical parameters including central tendency (e.g. means) or other basic estimates (e.g. regression coefficient) AND variation (e.g. standard deviation) or associated estimates of uncertainty (e.g. confidence intervals) |
| ☐ | ☒ | For null hypothesis testing, the test statistic (e.g. *F*, *t*, *r*) with confidence intervals, effect sizes, degrees of freedom and *P* value noted<br>*Give P values as exact values whenever suitable.* |
| ☒ | ☐ | For Bayesian analysis, information on the choice of priors and Markov chain Monte Carlo settings |
| ☒ | ☐ | For hierarchical and complex designs, identification of the appropriate level for tests and full reporting of outcomes |
| ☐ | ☒ | Estimates of effect sizes (e.g. Cohen's *d*, Pearson's *r*), indicating how they were calculated |

*Our web collection on statistics for biologists contains articles on many of the points above.*

## Software and code

Policy information about availability of computer code

| | |
|---|---|
| Data collection | Flow cytometry data was collected on FACSymphony A5 or LSR Fortessa (BD Biosciences) and sorting data on FACSAria Fusion Cell Sorter (BD Biosciences) or MA900 Multi-Application Cell Sorter (Sony Biotechnology). |
| Data analysis | Flow cytometry analyses were performed using FlowJo v.10.5.3 (BD Biosciences) and statistics assessed using Prism v.8.0.2 (GraphPad Software Inc.). Algorithms used for dimensionality reduction were UMAP (https://github.com/lmcinnes/umap) and Phenograph (https://github.com/ JinmiaoChenLab/Rphenograph). UMAP coordinates and Phenograph cluster annotation were assigned to each cell in each sample in the concatenated sample file and analyzed using FlowJo v.10.5.3 (BD Biosciences) and Prism v.8.0.2 (GraphPad Software Inc.). Circos software package version 0.69-9 (http://circos.ca/) was used for the circular plots. scRNAseq data analysis was performed in R v.3.5.2 (The R Foundation). Demultiplexing of the raw data and mapping to the human genome hg19 was done by the 10X CellRanger software (version 2.1.1; cellranger). QIAGEN Ingenuity Pathway Analysis tool was used for pathway analysis (Ingenuity Systems). The LAS X Life Science Microscope Software Leica Application Suite X version 3.0.1423224 (Leica Microsystems) was used for quantification pixels in double immunohistochemistry stainings. The acquired images by the MACSima™ Imaging Platform were preprocessed and analyzed using MACSiQ® View Imaging Software (Analysis Module; Milteny Biotec). |

For manuscripts utilizing custom algorithms or software that are central to the research but not yet described in published literature, software must be made available to editors and reviewers. We strongly encourage code deposition in a community repository (e.g. GitHub). See the Nature Portfolio guidelines for submitting code & software for further information.

# Data

Policy information about availability of data

All manuscripts must include a data availability statement. This statement should provide the following information, where applicable:

- Accession codes, unique identifiers, or web links for publicly available datasets
- A description of any restrictions on data availability
- For clinical datasets or third party data, please ensure that the statement adheres to our policy

All data associated with this study are available in the main text or the supplementary materials. scRNA-seq data have been deposited under accession number GSE169136 [https://www.ncbi.nlm.nih.gov/geo/query/acc.cgi?acc=GSE169136] and bulk RNA-seq data under accession number GSE171582 [https://www.ncbi.nlm.nih.gov/geo/query/acc.cgi?acc=GSE171582] in the Gene Expression Omnibus (GEO) database. Previously published data sets used in this paper are publicly available: GSE169749 [https://www.ncbi.nlm.nih.gov/geo/query/acc.cgi?acc=GSE169749] for murine spatial transcriptomics and GSE158328 [https://www.ncbi.nlm.nih.gov/geo/query/acc.cgi?acc=GSE158328] for human spatial transcriptomics. Source data are provided with this paper.

# Research involving human participants, their data, or biological material

Policy information about studies with human participants or human data. See also policy information about sex, gender (identity/presentation), and sexual orientation and race, ethnicity and racism.

Reporting on sex and gender

Sex was not considered in study design due to limited sample size.

Reporting on race, ethnicity, or other socially relevant groupings

Not relevant.

Population characteristics

Treatment naïve children undergoing diagnostic colonoscopy for suspected IBD as well as patients with already established IBD diagnosis were recruited, PBMCs and biopsies obtained the same day as the colonoscopy were used in the analysis. Inflamed and non-inflamed matched areas from the same individual were compared. After clinical and pathologic evaluation, which included clinical exam, radiology, laboratory tests, endoscopy and histopathology review, patients that were not diagnosed with IBD were considered as controls, and patients with other inflammatory or infectious diseases were excluded. For analyses across intestinal layers, colon tissue was collected from adults with IBD undergoing resection surgery at the Karolinska University Hospital as well as organ donors. For more details please see "Patients, samples, and disease scoring" section in the Methods, and Table S1 and S2 for patient characteristics.

For analyses across intestinal layers (flow cytometry), we used colon tissue from adult patients with IBD (n=3) undergoing resection surgery at the Karolinska University Hospital. Informed consents were obtained. The mean age was 45 years (standard deviation 30). Two of the three patients were female. For microscopy analysis, we used intestinal tissue from adult patients with IBD undergoing resection surgery at the Karolinska University Hospital (n=8) and organ donors (n=5). The mean age of organ donors was 68 years (standard deviation 12). One of the five patients were female. Based on macroscopic evaluation the samples from IBD patients included inflamed and non-inflamed areas, and this was further validated in the histopathology review. Eight were inflamed and three were non-inflamed, and the samples are anonymous.

Recruitment

Children undergoing first diagnostic colonoscopy for suspected IBD were recruited at the Department of Pediatric Gastroenterology, Hepatology and Nutrition, Karolinska University Hospital, Stockholm and Sachs' Children's and Youth Hospital, Södersjukhuset, Stockholm. Pinch biopsies were collected from these patients during routine colonoscopy and were matched with a blood sample for PBMC analysis, collected at the same day, prior to colonoscopy. After clinical and pathologic evaluation, which included clinical exam, radiology, laboratory tests, endoscopy and histopathology review, 48 patients were diagnosed with IBD and patients that were not diagnosed with IBD were considered as controls (n=17); patients with other inflammatory or infectious diseases were excluded. To control for mucosal inflammatory changes within the same patient, biopsies from a macroscopically inflamed area in colon were matched with a macroscopically non-inflamed or less inflamed area, as defined during the colonoscopy (macroscopic assessment). Next, this definition was further evaluated using histopathological review, and areas were finally confirmed to be either inflamed or non-inflamed (microscopic assessment) (Supplementary Fig. 1).
To assess cellular composition throughout the colon (caecum-ascendens-transversum-descendens-sigmoideum) under steady state and inflammation a unique clinical cohort was recruited to allow for matched comparisons: i) children that after endoscopy turned out to have an entirely healthy colon, ii) IBD patients with total colitis (meaning that the entire colon was equally inflamed).
For analyses across intestinal layers, colon tissue was collected from adults with IBD (n=8) undergoing resection surgery at the Karolinska University Hospital as well as organ donors (n=4).

Of note, pediatric patients were recruited at three largest centers for pediatric gastroenterology in the Stockholm region. Patients with suspected IBD in the childhood are referred to specialists in these centers, where the recruitment was performed. Since study cohorts were recruited at the largest centers for pediatric gastroenterology, where patients are treated by specialist pediatric gastroenterologists, the overall study population may include patients that have relatively more severe IBD than general population. Further details on recruitment are provided in the "Patients, samples, and disease scoring" section in the Methods.

Ethics oversight

The studies were approved by the Regional Review Board in Stockholm (2010/32-31/4, 2018/323-31/1, 2020-00507, 2019-05016), and written informed consents were obtained from patients and controls, as well as their parents for pediatric study participants.

Note that full information on the approval of the study protocol must also be provided in the manuscript.

# Field-specific reporting

Please select the one below that is the best fit for your research. If you are not sure, read the appropriate sections before making your selection.

☒ Life sciences ☐ Behavioural & social sciences ☐ Ecological, evolutionary & environmental sciences

For a reference copy of the document with all sections, see nature.com/documents/nr-reporting-summary-flat.pdf

# Life sciences study design

All studies must disclose on these points even when the disclosure is negative.

| | |
|---|---|
| Sample size | Sample size was determined based on prior experience from similar human studies, including rare pediatric samples (Kvedaraite et al, Gut 2016), as well as available resources and sample availability. No statistical methods were used to predetermine sample size number. |
| Data exclusions | No data was excluded. |
| Replication | All results shown were obtained from at least two separate experiments, using cells from different donors. |
| Randomization | Randomization was not relevant, because the study did not include any intervention for the patients. |
| Blinding | Blinding was applied in the following fashion: pediatric gastroenterologists who collected scored disease severity and diagnosed the patients did not analyze the immunological data. Blinding was not performed for the acquisition and analysis of the experimental data. Experimental observations would be consistent irrespective of blinding. |

# Reporting for specific materials, systems and methods

We require information from authors about some types of materials, experimental systems and methods used in many studies. Here, indicate whether each material, system or method listed is relevant to your study. If you are not sure if a list item applies to your research, read the appropriate section before selecting a response.

## Materials & experimental systems

| n/a | Involved in the study | |
|---|---|---|
| ☐ | ☒ | Antibodies |
| ☒ | ☐ | Eukaryotic cell lines |
| ☒ | ☐ | Palaeontology and archaeology |
| ☒ | ☐ | Animals and other organisms |
| ☒ | ☐ | Clinical data |
| ☒ | ☐ | Dual use research of concern |
| ☒ | ☐ | Plants |

## Methods

| n/a | Involved in the study | |
|---|---|---|
| ☒ | ☐ | ChIP-seq |
| ☐ | ☒ | Flow cytometry |
| ☒ | ☐ | MRI-based neuroimaging |

## Antibodies

Antibodies used

Anti-PDPN Unconjugated Clone NZ-1 OriGene Application IF/1:100, Clarity/1:10
Anti-αSMA Unconjugated Clone 1A4 Abcam Application Clarity/1:50
Goat Anti-Rat IgG AF488 Clone - Thermo Fisher Application IF/1:400, Clarity/1:100
Goat Anti-Mouse IgG2a AF594 Clone - Thermo Fisher Application Clarity/1:100
Anti-ICAM1 BV421 Clone HA58 BD Application FACS/1:50
Dead cell stain DCM aqua Clone - Thermo Fisher Application FACS/1:100
Anti-PDL1 BV605 Clone 29E.2A3 Biolegend Application FACS/1:50
Anti-CD11a BV650 Clone HI111 BD Application FACS/1:100
Anti-CD11b BV711 Clone ICRF44 Biolegend Application FACS/1:100
Anti-CD3 BV750 Clone SK7 Biolegend Application FACS/1:50
Anti-CD7 BV750 Clone M-T701 BD Application FACS/1:50
Anti-CD19 BV750 Clone HIB19 BD Application FACS/1:50
Anti-HLA-DR BV786 Clone L243 Biolegend Application FACS/1:50
Anti-CD206 BB515 Clone 19.2 BD Application FACS/1:50
Anti-CD1c BB700 Clone F10/21A3 BD Application FACS/1:50
Anti-CD31 PE-CF594 Clone WM59 Biolegend Application FACS/1:100
Anti-CD31 PB Clone WM59 Biolegend Application FACS/1:50
Anti-EPCAM PE-CF594 Clone 9C4 Biolegend Application FACS/1:800
Anti-CD123 PE-CY5 Clone 9F5 BD Application FACS/1:100
Anti-CD11c PE-Cy5.5 Clone BU15 Thermo Fisher Application FACS/1:200
Anti-PDPN PE-Cy7 Clone NC-08 Biolegend Application FACS/1:100

Anti-CLEC9A AF647 Clone 3A4 BD Application FACS/1:50
Anti-CD45 Alexa700 Clone HI30 BioLegend Application FACS/1:100
Anti-CD45 PE/CF594 Clone HI30 BD Application FACS/1:50
Anti-CD14 APC-Cy7 Clone M5E2 Biolegend Application FACS/1:200
Anti-CD14 BV570 Clone M5E2 Biolegend Application FACS/1:100
Anti-CD90 BUV395 Clone 5E10 BD Application FACS/1:400
Anti-CD90 BV605 Clone 5E10 BD Application FACS/1:50
Anti-CCR2 BUV615-P Clone LS132.1D9 BD Application FACS/1:100
Anti-CD15 BUV661 Clone W6D3 BD Application FACS/1:400
Anti-CD16 BUV737 Clone 3G8 BD Application FACS/1:400
Anti-CD16 BV785 Clone 3G8 Biolegend Application FACS/1:100
Anti-CD86 BUV805 Clone 2A9-1 BD Application FACS/1:25
Anti-Vimentin AF488 Clone RV202 BD Application FACS/1:50
Anti-CD19 PE-Cy5.5 Clone J3-119 Beckman Application FACS/1:100
Anti-IgG BUV563 Clone G18-145 BD Application FACS/1:200
Isotype control BUV563 Clone X40 BD Application FACS/1:200
Anti-aSMA AF488 Clone 1A4 R&D Application FACS/1:200
Anti-CD44 BV570 Clone IM7 Biolegend Application FACS/1:100
Anti-CD206 BV605 Clone 19.2 BD Application FACS/1:50
Anti-PDGFRA BV650 Clone αR1 BD Application FACS/1:25
Anti-HLA-DR BV711 Clone L243 Biolegend Application FACS/1:100
Anti-CD27 BV750 Clone O323 BD Application FACS/1:100
Anti-CD130 BB700 Clone AM64 BD Application FACS/1:100
Anti-CD146 PE Clone P1H12 Biolegend Application FACS/1:400
Anti-CD15 APC Clone W6D3 Biolegend Application FACS/1:100
Anti-CD56 BUV496 Clone NCAM16.2 BD Application FACS/1:100
Anti-CD38 BUV661 Clone G46-6 BD Application FACS/1:400
Anti-CD142 BUV737 Clone HTF-1 BD Application FACS/1:100
Anti-CD142 PE Clone HTF-1 LSBio Application FACS/1:50
Anti-CD9 BUV805 Clone M-L13 BD Application FACS/1:25
Anti-CD38 BV421 Clone HIT2 BD Application FACS/1:50
Anti-CD3 BV570 Clone UCHT1 Biolegend Application FACS/1:25
Anti-CD141 BV711 Clone 1A4 BD Application FACS/1:25
Anti-CD116 PE Clone 4H1 Biolegend Application FACS/1:50
Anti-PDL1 PE-CF594 Clone 29E.2A3 Biolegend Application FACS/1:400
Anti-CD4 PE-CY5 Clone OKT4 Biolegend Application FACS/1:200
Anti-FOLR2 APC Clone 94b/FOLR2 Biolegend Application FACS/1:200
Anti-CD123 BUV395 Clone 7G3 BD Application FACS/1:100
Anti-CD8 BUV496 Clone RPA-T8 BD Application FACS/1:25
Anti-CD5 BUV563 Clone UCHT2 BD Application FACS/1:50
Anti-CD15 BUV661 Clone W6D3 BD Application FACS/1:400

Antibodies for high-content imaging
Anti-CD206 EPR22489-7 Abcam ab254471 -
Anti-PDGFRA - Novus Biologicals AF-307-SP -
Anti-CCR2 K036C2 Biolegend 357207 APC
Anti-C3 EPR2988 Abcam ab196639 APC
Anti-PD-L1 28-8 Abcam ab224027 FITC
Anti-FOLR2 OTI4G6 FITC
Anti-CD163 EDHu-1 Novus Biologicals NB110-40686F FITC
Anti-PDGFRA_B Y92 Abcam ab196376 FITC
Anti-CD142_F3 HTF-1 LSBio LS-C751060 PE
Anti-CD146 EPR3208 Biolegend 361006 PE
Anti-CD123 REA918 Miltenyi Biotec 130-115-265 APC
Anti-CD45 5B1 Miltenyi Biotec 130-113-114 APC
Anti-CD209 (DC-SIGN) REAL690 Miltenyi Biotec 130-125-079 APC
Anti-CD15 VIMC6 Miltenyi Biotec 130-113-482 APC
Anti-CD235a REA175 Miltenyi Biotec 130-118-356 APC
Anti-CD3 REA1151 Miltenyi Biotec 130-120-269 APC
Anti-CD44 DB105 Miltenyi Biotec 130-113-331 APC
Anti-CD88 (C5AR) REA1213 Miltenyi Biotec 130-123-380 APC
Anti-CD20 Cytoplasmic REA543 Miltenyi Biotec 130-108-290 APC
Anti-CD8a REA1024 Miltenyi Biotec 130-117-202 APC
Anti-CD100 REA316 Miltenyi Biotec 130-104-600 FITC
Anti-CD1c (BDCA-1) REAL1005 Miltenyi Biotec 130-127-024 FITC
Anti-CD147 REA282 Miltenyi Biotec 130-124-221 FITC
Anti-CD52 REA164 Miltenyi Biotec 130-123-680 FITC
Anti-CD66b REA306 Miltenyi Biotec 130-123-694 FITC
Anti-CD45RA REA562 Miltenyi Biotec 130-113-365 FITC
Anti-Ki-67 REA183 Miltenyi Biotec 130-117-691 FITC
Anti-HLA-DR REAL550 Miltenyi Biotec 130-123-076 FITC
Anti-Myosin Smooth Muscle REA1107 Miltenyi Biotec 130-119-313 FITC
Anti-Cytokeratin REA831 Miltenyi Biotec 130-112-743 FITC
Anti-Actin (Smooth Muscle) REAL650 Miltenyi Biotec 130-123-363 FITC
Anti-CD279 (PD1) REA1165 Miltenyi Biotec 130-120-382 PE
Anti-CD234 (DARC) REA376 Miltenyi Biotec 130-125-845 PE

Anti-CD305 (LAIR-1) REA447 Miltenyi Biotec 130-126-091 PE
Anti-CD90 REAL677 Miltenyi Biotec 130-124-176 PE
Anti-CD38 REAL719 Miltenyi Biotec 130-126-438 PE
Anti-CD5 REAL760 Miltenyi Biotec 130-125-077 PE
Anti-PCNA REA858 Miltenyi Biotec 130-114-512 PE
Anti-Dectin-1 REA515 Miltenyi Biotec 130-121-993 PE
Anti-CD271 (LNGFR) REAL709 Miltenyi Biotec 130-125-053 PE
Anti-Caldesmon REA1120 Miltenyi Biotec 130-119-344 PE
Anti-CD13 REAL771 Miltenyi Biotec 130-125-787 PE
Anti-CD138 REA929 Miltenyi Biotec 130-115-479 PE
Anti-Jak1 REA700 Miltenyi Biotec 130-110-549 PE
Anti-HLA-DR, DP, DQ REA332 Miltenyi Biotec 130-120-715 PE
Anti-CD31 REA1312 Miltenyi Biotec 120-060-718 PE
Anti-CD14 REA1314 Miltenyi Biotec 120-060-722 PE
Anti-CD11b REA1321 Miltenyi Biotec 120-060-726 PE
Anti-CD68 REA1306 Miltenyi Biotec 120-060-257 PE
Anti-Calponin REA1104 Miltenyi Biotec 130-119-071 PE
Anti-Podoplanin REA446 Miltenyi Biotec 130-117-687 PE
Anti-Desmin REA1134 Miltenyi Biotec 130-119-490 PE
donkey Anti-goat - Invitrogen A16006 FITC
donkey Anti-rabbit - Invitrogen 12-4739-81 PE
rat Anti-mouse X-56 Miltenyi Biotec 130-119-585 PE

Validation

All antibodies are commercially available and are validated by the vendor on their official website.
Abcam: https://www.abcam.com/primary-antibodies/how-we-validate-our-antibodies
OriGene: https://www.origene.com/products/antibodies/primary-antibodies
BD Biosciences: https://www.bdbiosciences.com/en-eu/products/reagents/flow-cytometry-reagents/research-reagents/quality-and-reproducibility
Beckman Coulter: https://www.beckman.com/reagents/coulter-flow-cytometry/antibodies-and-kits/single-color-antibodies/quality-standards
Novus Biologicals: https://www.novusbio.com/reproducibility.html
Biolegend: https://www.biolegend.com/en-us/quality/quality-control
Miltenyi Biotec: https://www.miltenyibiotec.com/SE-en/products/macs-antibodies/antibody-validation.html
LSBio: https://www.lsbio.com/resources/ihc-antibody-validation
ThermoFisher Scientific: https://www.thermofisher.com/se/en/home/life-science/antibodies/invitrogen-antibody-validation.html
R&D: https://www.rndsystems.com/quality/antibodies-built-for-reproducibility

## Plants

Seed stocks

Not involved in the study

Novel plant genotypes

Not involved in the study

Authentication

Not involved in the study

## Flow Cytometry

### Plots

Confirm that:

☒ The axis labels state the marker and fluorochrome used (e.g. CD4-FITC).

☒ The axis scales are clearly visible. Include numbers along axes only for bottom left plot of group (a 'group' is an analysis of identical markers).

☒ All plots are contour plots with outliers or pseudocolor plots.

☒ A numerical value for number of cells or percentage (with statistics) is provided.

### Methodology

Sample preparation

To prepare cell suspensions, colonic tissue biopsies were digested using 250 µg/ml DNase and collagenase II (Sigma) at 37 °C with magnetic stirring at 650 rpm for 25 min, followed by filtering through a 70 µm cell strainer. Bacterial and fungal contamination was prevented by first incubating cell suspensions in PBS supplemented with 500 µg/ml Normocin (InvivoGen), 0,5 mg/ml gentamycin (Thermo Fisher Scientific) and 2,5 µg/ml Amphotericin B (Thermo Fisher Scientific) for 10 min shaking. Then, the cells were washed once, and resuspended and plated in DMEM supplemented with 10% FCS (Sigma), 2 mM L-glutamine (Thermo Fisher Scientific), 10 mM HEPES (Thermo Fisher Scientific), 1mM sodium pyruvate (Thermo Fisher

Scientific), 100 µg/ml Normocin (InvivoGen), 100 IU/ml penicillin (Thermo Fisher Scientific), and 100 µg/ml streptomycin (Thermo Fisher Scientific), further referred to as culture medium. After 2 h incubation at 37°C under a 5% $CO_2$ atmosphere, non-adherent cells were washed away and for the first passage culture medium was supplemented with 1 ng/ml PDGF-AB (Peprotech). Cells were then cultured for four to five passages until 80% confluence and sorted into PDPN+ and PDPN- fibroblast populations, collected in culture medium, instead of 10% supplemented with 30% FCS, after pre-gating on CD45-CD90+CD31-EPCAM- cells. PBMCs were isolated from blood samples after Ficoll separation using Lymphoprep (Stemcell Technologies). For FACS staining, cell suspensions were resuspended in PBS containing 2% FCS and 2 mM EDTA and a mixture of antibodies, supplemented with BD Horizon Brilliant Stain Buffer Plus (BD Biosciences) at 1:5 and FcR Blocking Reagent (Miltenyi Biotec) at 1:25 and were stained for 30 min.

Instrument

Cells were acquired on a FACSymphony A5 or LSR Fortessa (BD Biosciences) and sorted using FACSAria Fusion Cell Sorter (BD Biosciences) and MA900 Multi-Application Cell Sorter (Sony Biotechnology).

Software

FlowJo v.10.5.3 (BD Biosciences)

Cell population abundance

Cell purity was determined by flow cytometry and was more than 95%.

Gating strategy

Gating strategy for stromal cells is provided in Fig. 1, Fig. 2g, Supplementary Fig. 3b, 3d, and Supplementary Fig. 7a, 7b; for granulocytes in Supplementary Fig. 1a, immune cells in Supplementary Fig. 3b, 3e, 3f, for monocytes/macrophages from co-cultures in Supplementary Fig. 7c.

☒ Tick this box to confirm that a figure exemplifying the gating strategy is provided in the Supplementary Information.
